# Meiotic control of the APC/C: similarities & differences from mitosis

**DOI:** 10.1186/1747-1028-6-16

**Published:** 2011-08-01

**Authors:** Katrina F Cooper, Randy Strich

**Affiliations:** 1 University of Medicine and Dentistry of New Jersey, 2 Medical Center Drive, Stratford, NJ 08055, USA

## Abstract

The anaphase promoting complex is a highly conserved E3 ligase complex that mediates the destruction of key regulatory proteins during both mitotic and meiotic divisions. In order to maintain ploidy, this destruction must occur after the regulatory proteins have executed their function. Thus, the regulation of APC/C activity itself is critical for maintaining ploidy during all types of cell divisions. During mitotic cell division, two conserved activator proteins called Cdc20 and Cdh1 are required for both APC/C activation and substrate selection. However, significantly less is known about how these proteins regulate APC/C activity during the specialized meiotic nuclear divisions. In addition, both budding yeast and flies utilize a third meiosis-specific activator. In *Saccharomyces cerevisiae*, this meiosis-specific activator is called Ama1. This review summarizes our knowledge of how Cdc20 and Ama1 coordinate APC/C activity to regulate the meiotic nuclear divisions in yeast.

## Meiosis and gametogenesis

**Figure 1:**
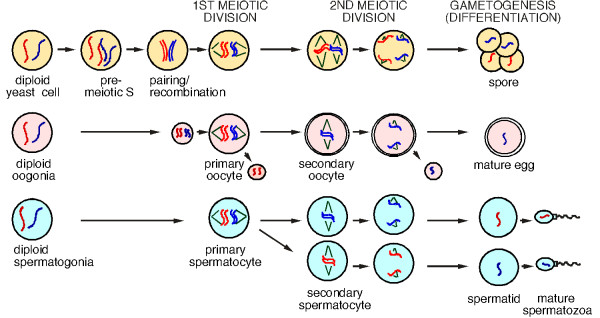
**Meiotic divisions are conserved between yeast and higher eukaryotes including mammals**. Cartoon showing the similarities between the meiotic divisions in yeast and mammals. The red and the blue lines represent chromosomes. Pre-meiotic S, pairing and recombination occur in oogenesis and spermatogenesis but have only been drawn for meiosis in yeast for clarity.

The proper segregation of chromosomes at meiosis I and II is essential for producing gametes with the correct haploid genome (Figure 1). During oogenesis, meiotic progression is arrested at the first or second division during development. Maturation of the oocytes or fertilization is required to relieve these blocks, respectively. Spermatogenesis is a continuous process that occurs throughout most of the life of the male. Yeast sporulation possesses the hallmarks of mammalian meiosis and is similar to spermatogenesis in that the process does not exhibit programmed arrest points. In *Saccharomyces cerevisiae*, entry into the meiotic program is dependent upon cell-type and environmental clues [1]. Following induction, premeiotic DNA replication occurs followed by a lengthy prophase in which homologous chromosomes synapse and undergo a high level of genetic recombination prior to meiosis I ([2] & Figure 1). This genetic exchange is essential for chromosomes to correctly align at metaphase I. It is during meiosis I, the reductional division, that the sister chromatids remain paired, attach to only one spindle, and segregate together. This centromeric cohesion is lost during the second meiotic division, which resembles mitosis, where the replicated sisters make bipolar attachments and separate to opposite poles [3]. The resulting four haploid nuclei are each encased in a multi-layered structure called a spore that remains dormant until induced to reenter mitotic cell division by growth signals [1]. Thus, the monopolar attachment of replicated sister chromatids at meiosis I and the execution of two nuclear divisions without an intervening S phase represent two major differences between meiotic and mitotic divisions.

## Specialized control of mitotic cell cycle machinery required for meiotic nuclear divisions

The basic cell cycle machinery driving mitotic cell division (e.g., DNA polymerases, cyclin dependent kinases, ubiquitin ligases) is also required to execute meiosis. However, meiosis presents several challenges that are not found during mitosis such as maintaining sister chromatid attachment during the reductional division or undergoing two nuclear divisions without an intervening S phase. Studies in *S. cerevisiae *have identified two strategies by which the mitotic cell cycle machinery is redirected to execute the meiotic divisions. The first method involves replacing mitotic regulatory proteins with meiotic counterparts. For example, Rec8 replaces Mcd1 to maintain sister centromere cohesion during meiosis I [4]. In addition, Ama1 is a meiosis-specific activator of the anaphase promoting complex/cyclosome (APC/C) ubiquitin ligase and is required for exit from meiosis II [5-8]. The second approach utilizes mitotic regulators that take on new meiotic functions. For example, the mitotic S-phase cyclins Clb5 and Clb6 are required for the initiation of recombination and synaptoneal complex formation during meiosis [9]. Furthermore, the APC/C^Cdc20 ^ubiquitin ligase that controls the G2/M transition in mitotic cells also has a meiosis-specific role to induce early meiotic gene transcription as well as progression through prophase I [8,10,11]. The focus of this review is to summarize our knowledge of how the APC/C regulates, and how it is regulated by, the meiotic differentiation program in the model system *S. cerevisiae*.

## Role of APC/C activators during mitotic division

**Figure 2:**
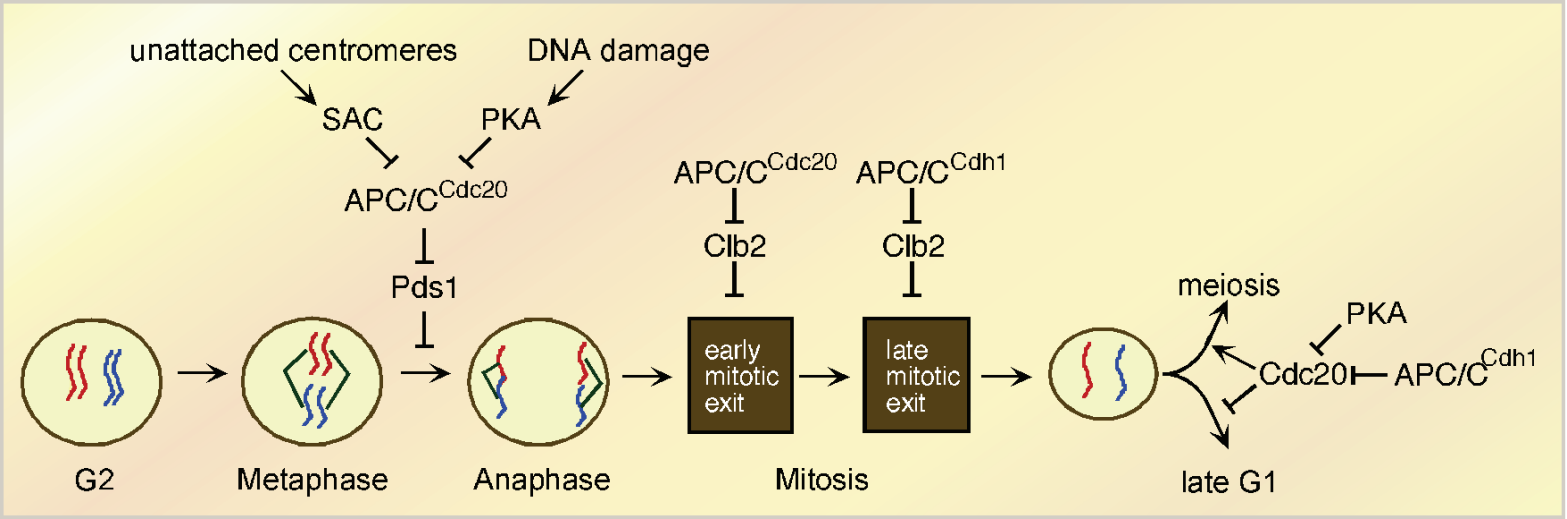
**Regulation of the G2/M transition and mitotic exit by the APC/C**. Destruction of Pds1 (securin) by APC/C^Cdc20 ^triggers the metaphase-anaphase transition. Checkpoint pathways monitoring spindle attachment or DNA damage can inhibit APC/C^Cdc20 ^activity by direct association of spindle assembly checkpoint (SAC) components or phosphorylation by PKA. The exit from mitosis initially requires the degradation of several regulatory proteins including the B-type cyclin Clb2 by APC/C^Cdc20^. Final mitotic exit requires APC/C^Cdh1 ^which continues Clb2 degradation to completion. APC/C^Cdh1 ^remains active in G1 partially destroying Cdc20. The decision to enter meiosis occurs early in G1 and requires APC/C^Cdc20 ^destruction of Ume6. Inhibition of Cdc20 function by PKA phosphorylation drives the cell through G1 to reinitiate another round of mitotic cell division.

To examine the regulation and activity of APC/C^Cdc20 ^during meiosis, it is helpful to first start with what is known about this ligase's function and regulation during mitotic cell division. The APC/C is a multi-subunit ubiquitin ligase that directs the destruction of cell cycle regulatory proteins at the metaphase-anaphase transition, exit from mitosis, and G1 [12]. The control of APC/C activity and specificity is complex (for reviews see [13-16]). During mitotic cell division, APC/C activation depends on its sequential association with two evolutionarily conserved coactivators, Cdc20 and Cdh1 (Figure 2). In brief, in the presence of high cyclin dependent kinase (Cdk) activity, Cdc20 activated APC/C (APC/C^Cdc20^) promotes the metaphase-anaphase transition by directing the destruction of the anaphase inhibitor Pds1/securin [17-20] causing subsequent dissolution of the cohesin complex holding the sister chromatids together (see [21] and references therein). After anaphase, APC/C^Cdh1 ^mediates the final degradation of mitotic B-type cyclins and several other proteins [22-27] as the cell exits mitosis and enters G1. In S phase and G2, the APC/C is inactive to allow accumulation of proteins required for building the mitotic spindle.

## Regulation of Cdc20 during mitotic cell division

APC/C mediated proteolysis of key regulatory proteins drives the cell from G2 through M phase into G1. Accordingly, the APC/C is under a strict temporal control so these targets are destroyed in the correct order. Toward this end, APC/C^Cdc20 ^is regulated by at least four mechanisms. First, Cdc20 levels are modulated by transient transcription from S phase through G2 phase and proteolysis in G1 [28,29]. Once associated, APC/C^Cdc20 ^is inhibited in G2 by Mad2p, a component of the spindle assembly checkpoint (SAC) pathway [30-32] (Figure 2). In addition, activation of the DNA damage checkpoint pathway inhibits Cdc20 activity by direct phosphorylation by Protein Kinase A (PKA) [33]. Achieving bi-polar attachment of chromosomes on the metaphase plate extinguishes the spindle checkpoint signal permitting securin (Pds1) ubiquitylation/destruction and anaphase to proceed [34]. A unified molecular model of how checkpoint proteins block APC-mediated ubiquitylation of securin has not been established. Recently, Mad3 has emerged as a key player in this process that both mediates Cdc20 degradation in prometaphase by an unknown mechanism [35-37] and acts as an APC/C pseudo-substrate inhibitor [38]. In G1, APC/C^Cdh1 ^and a proteasome independent mechanism induce Cdc20 proteolysis as the cells prepare for the initiation of DNA replication [28]. In addition to proteolysis, Cdc20 is again negatively regulated by PKA but at a different site to prevent the initiation of meiosis (see below).

## APC/C^Cdc20 ^activity is required for entry into the meiotic program

**Figure 3:**
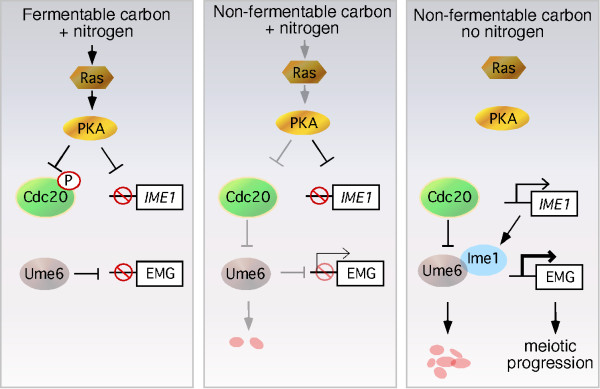
**APC/C^Cdc20 ^mediated destruction of Ume6 is required for meiotic entry**. Under rich growing conditions, PKA phosphorylation inhibits Cdc20 activity both protecting destruction of Ume6 is required for meiotic entry and preventing transcription of the meiotic inducer *IME1* (left panel). Switching cultures to medium lacking a fermentable carbon source but containing nitrogen reduces PKA activity which permits partial Ume6 destruction (middle panel). Removing nitrogen allows Ime1 production which, along with fully active Cdc20, completely destroys Ume6 allowing early meiotic gene (EMG) induction and meiotic progression (right panel).

To enter the meiotic program, cells exit the cell cycle early in G1 before the accumulation of the G1 cyclins [39]. The transition between mitotic and meiotic cell division requires the destruction of the transcriptional repressor Ume6 by APC/C^Cdc20 ^[10]. Ume6 is a C6 zinc cluster DNA binding protein [40] that represses early meiotic genes during mitotic cell division in the presence of nitrogen and a fermentable carbon source (Figure 3, left panel). Under rich growth conditions, activated PKA phosphorylation of Cdc20 (at a site different than targeted following DNA damage) restricts APC/C activity, possibly by preventing the interaction of Cdc20 with some of its substrates [33,41]. This model is consistent with the observation that Cdc20 and Ume6 do not associate under rich growth conditions [10]. Ume6 destruction has been divided into a two-step process. The first step partially degrades Ume6 and occurs in cultures growing in medium containing nitrogen and only a non-fermentable carbon source (Figure 3, middle panel). In this medium, PKA activity is reduced along with the inhibitory phosphorylation on Cdc20. This reduction in Ume6 levels results in a low level derepression of early meiotic genes. However, Ume6 destruction is not complete until cells are shifted to media lacking both nitrogen and a fermentable carbon source (Figure 3, right panel). Under these conditions, the *IME1 *gene is transcribed and the association of its gene product with Ume6 completes APC/C^Cdc20 ^dependent destruction [10]. Once Ume6 destruction is complete, EMG transcription is induced and meiotic program is initiated. The mechanism for how Ime1 association mediates the final destruction of Ume6 is not known. However the presence of Ime1 stimulates Ume6 ubiquitylation by APC/C^Cdc20 ^in vitro (unpublished results). These findings suggest a model that APC/C^Cdc20 ^is re-tasked by the presence of Ime1 to complete Ume6 destruction. Recent studies indicate that APC/C regulation of post-mitotic differentiation programs may be more common than previously appreciated (reviewed in [42]). For example, the oncoprotein Sno, a negative regulator of the SMAD pathway, is destroyed in an APC/C dependent manner following TGFβ stimulation [43]. In addition, a post-mitotic role for the APC/C has been observed in neurons [43,44]. Finally, in a system perhaps analogous to APC/C^Cdc20 ^and Ume6, destruction of the transcriptional repressor Id2 by APC/C^Cdh1 ^is required for exit from the mitotic cell cycle and to restrain axonal growth in neurons [45]. Therefore, the introduction of a developmentally regulated protein such as Ime1 may provide a mechanism by which the substrate spectrum of the APC/C can be altered in the context of a differentiation program.

## APC/C^Cdc20 ^is required for both meiotic divisions

**Figure 4:**
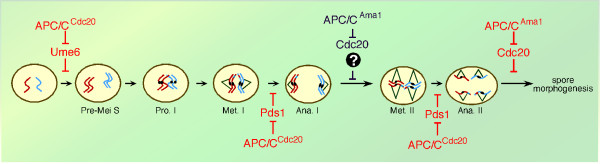
**Regulation of meiotic progression by the APC/C**. Diagram showing the known (red) and potential (purple) execution points for APC/C^Cdc20 ^and APC/C^Ama1 ^activity during meiosis.

Evidence from many groups indicate that APC/C^Cdc20 ^triggers Pds1/Securin destruction prior to each nuclear division (Figure 4). For example, temperature sensitive *cdc20 *mutants arrest at prophase I when cells are shifted to the restrictive temperature after meiotic entry [8,11]. In addition, wild-type cells expressing a non-destructible allele of *PDS1 *also arrest at prophase I [46-48]. Lastly, single cell immunofluorescence studies revealed Pds1 proteolysis prior to both meiotic divisions [11]. Surprisingly, APC/C^Ama1 ^also has the capability to destroy Pds1 during the meiotic divisions [47,49]. However, this destruction only occurs in cells lacking the APC/C inhibitor Mnd2 [5,47,49].

## Multiple mechanisms regulate APC/C^Cdc20 ^activity during meiosis

**Figure 5:**
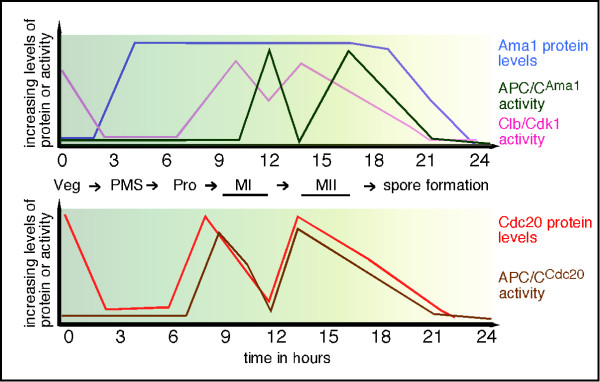
**Regulation of Ama1 and Cdc20 activity during meiosis**. The upper graph depicts the relationship between APC/C^Ama1 ^activity and Ama1 protein accumulation during meiosis. In addition, Clb/Cdk1 activity is presented. The bottom graph illustrates the relationship between APC/C^Cdc20 ^activity and Cdc20 protein accumulation during meiosis.

The role for APC/C^Cdc20 ^in both nuclear divisions imply that its activity must oscillate during this stage in development. Specifically, APC/C^Cdc20 ^must be inactive to permit Pds1 accumulation at metaphase I, activated to destroy it at anaphase I, then toggle off and on again to allow the second division to occur (Figure 5). *CDC20 *expression is under the control of the *NDT80 *transcription factor and its mRNA is present during both meiotic divisions [8,50]. Using the presence or absence of an indirect immunofluorescence signal, Cdc20 levels were reported to dramatically fall between anaphase I and metaphase II [11] suggesting that protein destruction represented a key regulatory strategy. A potential clue for how Cdc20 levels are modulated came from the finding that Cdc20 is destroyed by APC/C^Ama1 ^as cell exit meiosis II [8]. This result was different than G1 mitotic cells which utilize a combination of APC/C dependent and independent mechanisms to accomplish this task. Interestingly, Mnd2-dependent inhibition of APC/C^Ama1 ^is mitigated prior to anaphase I, consistent with a role in Cdc20 degradation prior to metaphase II (Figure 4). However, it is not clear how APC/C^Ama1 ^activity (Figure 5) would then be inhibited to allow subsequent accumulation of Cdc20 necessary for execution of the second division. In *S. pombe*, as well as higher eukaryotes, the APC/C is inhibited at the MI/MII transition by specific endogenous inhibitors (reviewed in [51]). Therefore, one possibility is that a meiosis-specific inhibitor is synthesized to transiently curtail APC/C^Ama1 ^activity. Interestingly, cells deleted for *cdh1 *fail to induce Cdc20 during meiosis yet display similar execution kinetics and spore viability as wild type [8]. This suggests a model in which Cdh1 is indirectly required to keep Ama1 inactive until cells reach anaphase I exit.

## Regulation of Cdc20 as cells exit meiosis

Upon exit from the second meiotic division, APC/C^Ama1 ^mediates Cdc20 destruction through two degrons, a destruction box and a GxEN element [8]. In *S. cerevisiae*, Cdc20 destruction is not essential for meiotic progression as introducing a stabilized allele of *CDC20*, under the control of the Ama1 promotor, did not affect spore production or viability [8]. This result suggests that APC/C^Cdc20 ^can be inactivated by alternative mechanisms. For example, dephosphorylation of core APC/C subunits, possibly by PP1 or PP2A phosphatases, decreases APC/C activity (reviewed in [14]). In support of this idea, dephosphorylation of Cdc20 is important for release from metaphase II arrest in Xenopus egg extracts [52,53]. APC/C inactivation at the end meiosis is also critical for embryonic development in *Drosophila *[54]. Here, the meiosis-specific APC/C activator CORT (also known as CORTEX, [55]) is destroyed by APC/C^FZY ^(Cdc20p homologue) by completion of meiosis in the early embryo. Moreover, this degradation is destruction box dependent and hypothesized to be important for embryogenesis [54].

Finally, one interesting mechanistic question is how Cdc20 switches from being an activator to a substrate of the APC/C. Extensive studies have been devoted to a molecular understanding of APC/C substrate and activator recognition in mitotically dividing cells (reviewed in [56-58]). It is known that the conserved APC/C binding motifs (called C-box and IR motif) are required for APC/C binding of Cdc20, Cdh1 and Ama1 [8,59,60]. Cdc20 binding to the APC/C via these motifs is not required for its destruction [8]. This suggests a model in which once Cdc20 is dissociated from the core APC/C, it is targeted for degradation by APC/C^Ama1^.

## Concluding remarks

It is clear that regulatory system governing meiotic development borrowed heavily from the system controlling mitotic cell division. For example, targeted ubiquitin mediated proteolysis of key regulatory factors still pushes meiosis and mitosis in one direction. In addition, these destruction pathways are governed by checkpoint surveillance systems to ensure the execution of one event before proceeding to the next. However, unique characteristics associated with meiosis such as haploidization, and the fact that meiosis is not a cycle but a linear differentiation pathway, necessitated significant modification of the mitotic regulatory pathways. At the onset, APC/C^Cdc20^-dependent destruction of Ume6 sits at the decision point between meiosis and mitosis. Destroying Ume6 induces a specialized set of genes able to induce meiS phase under conditions (absence of nitrogen and other nutrients) that would prohibit mitotic S phase. Next, the ability to execute two nuclear divisions without an intervening S phase requires delicate fine tuning of APC/C^Cdc20 ^activity to permit reassembly of the meiosis II spindle without allowing formation of the pre-replication complex on DNA replication origins. Finally, as post-meiotic cells can be dormant for extended time periods, the destruction of all three APC/C activators protects against precocious re-entry into the mitotic cell cycle.

## Competing interests

The authors declare that they have no competing interests.

## Authors' contributions

KFC and RS wrote the manuscript together. Both authors read and approved the final manuscript.

## References

[B1] Kupiec M Byers B Esposito RE Mitchell AP Pringle JR, Broach JR, Jones EW Meiosis and sporulation in Saccharomyces cerevisiae The molecular and cellular biology of the yeast Saccharomyces 1997 Cold Spring Harbor, NY: Cold Spring Harbor Press 889 1036

[B2] Szekvolgyi L Nicolas A From meiosis to postmeiotic events: homologous recombination is obligatory but flexible Febs J 2010 277 571 589 10.1111/j.1742-4658.2009.07502.x 20015080

[B3] Sakuno T Watanabe Y Studies of meiosis disclose distinct roles of cohesion in the core centromere and pericentromeric regions Chromosome Res 2009 17 239 249 10.1007/s10577-008-9013-y 19308704

[B4] Klein F Mahr P Galova M Buonomo SB Michaelis C Nairz K Nasmyth K A central role for cohesins in sister chromatid cohesion, formation of axial elements, and recombination during yeast meiosis Cell 1999 98 91 103 10.1016/S0092-8674(00)80609-1 10412984

[B5] Cooper KF Egeland DE Mallory MJ Jarnik M Strich R Ama1p is a Meiosis-Specific Regulator of the Anaphase Promoting Complex/Cyclosome in yeast Proc Natl Acad Sci USA 2000 97 14548 14553 10.1073/pnas.250351297 11114178 PMC18956

[B6] McDonald CM Cooper KF Winter E The Ama1-Directed Anaphase-Promoting Complex Regulates the Smk1 Mitogen-Activated Protein Kinase During Meiosis in Yeast Genetics 2005 171 901 911 10.1534/genetics.105.045567 16079231 PMC1456836

[B7] Diamond AE Park JS Inoue I Tachikawa H Neiman AM The anaphase promoting complex targeting subunit Ama1 links meiotic exit to cytokinesis during sporulation in Saccharomyces cerevisiae Mol Biol Cell 2009 20 134 145 10.1091/mbc.E08-06-0615 18946082 PMC2613089

[B8] Tan GS Magurno J Cooper KF Ama1p-activated anaphase-promoting complex regulates the destruction of Cdc20p during meiosis II Mol Biol Cell 2011 22 315 326 10.1091/mbc.E10-04-0360 21118994 PMC3031463

[B9] Smith KN Penkner A Ohta K Klein F Nicolas A B-type cyclins CLB5 and CLB6 control the initiation of recombination and synaptonemal complex formation in yeast meiosis Curr Biol 2001 11 88 97 10.1016/S0960-9822(01)00026-4 11231124

[B10] Mallory MJ Cooper KF Strich R Meiosis-specific destruction of the Ume6p repressor by the Cdc20-directed APC/C Mol Cell 2007 27 951 961 10.1016/j.molcel.2007.08.019 17889668 PMC2034308

[B11] Salah SM Nasmyth K Destruction of the securin Pds1p occurs at the onset of anaphase during both meiotic divisions in yeast Chromosoma 2000 109 27 34 10.1007/s004120050409 10855492

[B12] Peters JM SCF and APC: the Yin and Yang of cell cycle regulated proteolysis Curr Opin Cell Biol 1998 10 759 768 10.1016/S0955-0674(98)80119-1 9914180

[B13] Tyers M Jorgensen P Proteolysis and the cell cycle: with this RING I do thee destroy Curr Opin Genet Dev 2000 10 54 64 10.1016/S0959-437X(99)00049-0 10679394

[B14] Harper JW Burton JL Solomon MJ The anaphase-promoting complex: it's not just for mitosis any more Genes & development 2002 16 2179 2206 10.1101/gad.1013102 12208841

[B15] Peters JM The anaphase promoting complex/cyclosome: a machine designed to destroy Nat Rev Mol Cell Biol 2006 7 644 656 10.1038/nrm1988 16896351

[B16] Wasch R Robbins JA Cross FR The emerging role of APC/CCdh1 in controlling differentiation, genomic stability and tumor suppression Oncogene 2010 29 1 10 19826416 10.1038/onc.2009.325 PMC3102600

[B17] Kramer ER Scheuringer N Podtelejnikov AV Mann M Peters JM Mitotic regulation of the APC activator proteins CDC20 and CDH1 [In Process Citation] Mol Biol Cell 2000 11 1555 1569 10793135 10.1091/mbc.11.5.1555 PMC14867

[B18] Rudner AD Murray AW Phosphorylation by cdc28 activates the Cdc20-dependent activity of the anaphase-promoting complex J Cell Biol 2000 149 1377 1390 10.1083/jcb.149.7.1377 10871279 PMC2175139

[B19] Visintin R Prinz S Amon A *CDC20 *and *CDH1*: a family of substrate-specific activators of APC-dependent proteolysis Science 1997 278 460 463 10.1126/science.278.5337.460 9334304

[B20] Cohen-Fix O Koshland D The anaphase inhibitor of Saccharomyces cerevisiae Pds1p is a target of the DNA damage checkpoint pathway Proc Natl Acad Sci USA 1997 94 14361 14366 10.1073/pnas.94.26.14361 9405617 PMC24978

[B21] Nasmyth K Haering CH Cohesin: its roles and mechanisms Annual review of genetics 2009 43 525 558 10.1146/annurev-genet-102108-134233 19886810

[B22] Schwab M Schulze Lutum A Seufert W Yeast Hct1 is a regulator of Clb2 cyclin protolysis Cell 1997 90 683 693 10.1016/S0092-8674(00)80529-2 9288748

[B23] Shirayama M Zachariae W Ciosk R Nasmyth K The Polo-like kinase Cdc5p and the WD-repeat protein Cdc20p/fizzy are regulators and substrates of the anaphase promoting complex in *Saccharomyces cerevisiae* The EMBO journal 1998 17 1336 1349 10.1093/emboj/17.5.1336 9482731 PMC1170482

[B24] Hildebrandt ER Hoyt MA Cell cycle-dependent degradation of the Sacharomyces cerevisae spindle motor Cin8p requires APCCdh1 and a bipartite destruction sequence Mol Biol Cell 2001 12 3402 3416 11694576 10.1091/mbc.12.11.3402 PMC60263

[B25] Woodbury EL Morgan DO Cdk and APC activities limit the spindle-stabilizing function of Fin1 to anaphase Nature cell biology 2007 9 106 112 10.1038/ncb1523 17173039

[B26] Juang Y-L Huang J Peters J-M McLaughlin ME Tai C-Y Pellman D APC-mediated proteolysis of Ase1 and the morphogenesis of the mitotic spindle Science 1997 275 1311 1314 10.1126/science.275.5304.1311 9036857

[B27] Crasta K Huang P Morgan G Winey M Surana U Cdk1 regulates centrosome separation by restraining proteolysis of microtubule-associated proteins The EMBO journal 2006 25 2551 2563 10.1038/sj.emboj.7601136 16688214 PMC1478175

[B28] Huang JN Park I Ellingson E Littlepage LE Pellman D Activity of the APCCdh1 form of the anaphase promoting complex persists until S phase and prevents premature expression of Cdc20p The Journal of biological chemistry 2001 154 85 94 10.1083/jcb.200102007 PMC2196868 11448992

[B29] Fang G Yu H Kirschner MW Direct binding of CDC20 protein family members activates the anaphase-promoting complex in mitosis and G1 Molecular Cell 1998 2 163 171 10.1016/S1097-2765(00)80126-4 9734353

[B30] Minshull J Sun H Tonks NK Murray AW A MAP kinase-dependent spindle assembly checkpoint in Xenopus egg extracts Cell 1994 79 475 486 10.1016/0092-8674(94)90256-9 7954813

[B31] Hwang LH Lau LF Smith DL Mistrot CA Hardwick KG Hwang ES Amon A Murray AW Budding yeast Cdc20: a target of the spindle checkpoint Science 1998 279 1041 1044 10.1126/science.279.5353.1041 9461437

[B32] Zhang Y Lees E Identification of an overlapping binding domain on Cdc20 for Mad2 and anaphase-promoting complex: model for spindle checkpoint regulation Mol Cell Biol 2001 21 5190 5199 10.1128/MCB.21.15.5190-5199.2001 11438673 PMC87243

[B33] Searle JS Schollaert KL Wilkins BJ Sanchez Y The DNA damage checkpoint and PKA pathways converge on APC substrates and Cdc20 to regulate mitotic progression Nature cell biology 2004 6 138 145 10.1038/ncb1092 14743219

[B34] Logarinho E Bousbaa H Kinetochore-microtubule interactions "in check" by Bub1, Bub3 and BubR1: The dual task of attaching and signalling Cell cycle (Georgetown, Tex) 2008 7 1763 1768 10.4161/cc.7.12.6180 18594200

[B35] Hardwick KG Johnston RC Smith DL Murray AW MAD3 encodes a novel component of the spindle checkpoint which interacts with Bub3p, Cdc20p, and Mad2p J Cell Biol 2000 148 871 882 10.1083/jcb.148.5.871 10704439 PMC2174553

[B36] Pan J Chen RH Spindle checkpoint regulates Cdc20p stability in Saccharomyces cerevisiae Genes & development 2004 18 1439 1451 10.1101/gad.1184204 15198982 PMC423194

[B37] King EM van der Sar SJ Hardwick KG Mad3 KEN boxes mediate both Cdc20 and Mad3 turnover, and are critical for the spindle checkpoint PloS one 2007 2 e342 10.1371/journal.pone.0000342 17406666 PMC1829190

[B38] Burton JL Solomon MJ Mad3p, a pseudosubstrate inhibitor of APCCdc20 in the spindle assembly checkpoint Genes & development 2007 21 655 667 10.1101/gad.1511107 17369399 PMC1820940

[B39] Colomina N Gari E Gallego C Herrero E Aldea M G1 cyclins block the Ime1 pathway to make mitosis and meiosis incompatible in budding yeast The EMBO journal 1999 18 320 329 10.1093/emboj/18.2.320 9889189 PMC1171127

[B40] Strich R Surosky RT Steber C Dubois E Messenguy F Esposito RE UME6 is a key regulator of nitrogen repression and meiotic development Genes & development 1994 8 796 810 10.1101/gad.8.7.796 7926768

[B41] Bolte M Dieckhoff P Krause C Braus GH Irniger S Synergistic inhibition of APC/C by glucose and activated Ras proteins can be mediated by each of the Tpk1-3 proteins in Saccharomyces cerevisiae Microbiology (Reading, England) 2003 149 1205 1216 10.1099/mic.0.26062-0 12724382

[B42] Qiao X Zhang L Gamper AM Fujita T Wan Y APC/C-Cdh1: from cell cycle to cellular differentiation and genomic integrity Cell cycle (Georgetown, Tex) 2010 9 3904 3912 10.4161/cc.9.19.13585 PMC3047751 20935501

[B43] Teng FY Tang BL APC/C regulation of axonal growth and synaptic functions in postmitotic neurons: the Liprin-alpha connection Cell Mol Life Sci 2005 10.1007/s00018-005-5043-1 PMC11139192 15924262

[B44] van Roessel P Elliott DA Robinson IM Prokop A Brand AH Independent regulation of synaptic size and activity by the anaphase-promoting complex Cell 2004 119 707 718 10.1016/j.cell.2004.11.028 15550251

[B45] Lasorella A Stegmuller J Guardavaccaro D Liu G Carro MS Rothschild G de la Torre-Ubieta L Pagano M Bonni A Iavarone A Degradation of Id2 by the anaphase-promoting complex couples cell cycle exit and axonal growth Nature 2006 442 471 474 10.1038/nature04895 16810178

[B46] Shonn MA McCarroll R Murray AW Requirement of the spindle checkpoint for proper chromosome segregation in budding yeast meiosis Science 2000 289 300 303 10.1126/science.289.5477.300 10894778

[B47] Oelschlaegel T Schwickart M Matos J Bogdanova A Camasses A Havlis J Shevchenko A Zachariae W The yeast APC/C subunit Mnd2 prevents premature sister chromatid separation triggered by the meiosis-specific APC/C-Ama1 Cell 2005 120 773 788 10.1016/j.cell.2005.01.032 15797379

[B48] Cooper KF Mallory MJ Guacci V Lowe K Strich R Pds1p is required for meiotic recombination and prophase I progression in Saccharomyces cerevisiae Genetics 2009 181 65 79 19001291 10.1534/genetics.108.095513 PMC2621190

[B49] Penkner AM Prinz S Ferscha S Klein F Mnd2, an essential antagonist of the anaphase-promoting complex during meiotic prophase Cell 2005 120 789 801 10.1016/j.cell.2005.01.017 15797380

[B50] Chu S DeRisi J Eisen M Mulholland J Botstein D Brown PO Herskowitz I The transcriptional program of sporulation in budding yeast Science 1998 282 699 705 9784122 10.1126/science.282.5389.699

[B51] Pesin JA Orr-Weaver TL Regulation of APC/C activators in mitosis and meiosis Annu Rev Cell Dev Biol 2008 24 475 499 10.1146/annurev.cellbio.041408.115949 18598214 PMC4070676

[B52] Yudkovsky Y Shteinberg M Listovsky T Brandeis M Hershko A Phosphorylation of Cdc20/fizzy negatively regulates the mammalian cyclosome/APC in the mitotic checkpoint Biochemical and biophysical research communications 2000 271 299 304 10.1006/bbrc.2000.2622 10799291

[B53] Costanzo M Nishikawa JL Tang X Millman JS Schub O Breitkreuz K Dewar D Rupes I Andrews B Tyers M CDK activity antagonizes Whi5, an inhibitor of G1/S transcription in yeast Cell 2004 117 899 913 15210111 10.1016/j.cell.2004.05.024

[B54] Pesin JA Orr-Weaver TL Developmental role and regulation of cortex, a meiosis-specific anaphase-promoting complex/cyclosome activator PLoS Genet 2007 3 e202 10.1371/journal.pgen.0030202 18020708 PMC2077894

[B55] Chu T Henrion G Haegeli V Strickland S Cortex, a Drosophila gene required to complete oocyte meiosis, is a member of the Cdc20/fizzy protein family Genesis 2001 29 141 152 10.1002/gene.1017 11252055

[B56] Matyskiela ME Morgan DO Analysis of activator-binding sites on the APC/C supports a cooperative substrate-binding mechanism Mol Cell 2009 34 68 80 10.1016/j.molcel.2009.02.027 19362536 PMC2754851

[B57] Pines J The APC/C: a smorgasbord for proteolysis Mol Cell 2009 34 135 136 10.1016/j.molcel.2009.04.006 19394289

[B58] Yu H Cdc20: a WD40 activator for a cell cycle degradation machine Mol Cell 2007 27 3 16 10.1016/j.molcel.2007.06.009 17612486

[B59] Schwab M Neutzner M Mocker D Seufert W Yeast Hct1 recognizes the mitotic cyclin Clb2 and other substrates of the ubiquitin ligase APC The EMBO journal 2001 20 5165 5175 10.1093/emboj/20.18.5165 11566880 PMC125620

[B60] Vodermaier HC Gieffers C Maurer-Stroh S Eisenhaber F Peters JM TPR subunits of the anaphase-promoting complex mediate binding to the activator protein CDH1 Curr Biol 2003 13 1459 1468 10.1016/S0960-9822(03)00581-5 12956947

